# Pediatric Gastroenterology—challenges great and small

**DOI:** 10.3389/fped.2013.00002

**Published:** 2013-02-21

**Authors:** Andrew S. Day

**Affiliations:** Department of Pediatrics, University of Otago (Christchurch)Christchurch, New Zealand

## Introduction

Pediatric Gastroenterology encompasses one of the broadest pediatric subspecialities. Areas of interest include luminal conditions, hepatology, pancreatic diseases, and nutritional disorders, along with interactions between these (such as luminal disorders disrupting nutrition). Furthermore, pediatric gastroenterology services regularly intersect with almost all other subspecialities.

Pediatric gastroenterology faces many different challenges. These include the type and character of the conditions cared for, the changing patterns of conditions over time, new developments and technologies, and expanding knowledge. Some of these particular challenges will be highlighted here.

## Chronic or incurable conditions

Many of the important conditions cared for by pediatric gastroenterologists are chronic or incurable, and impact not just during the child's initial years but throughout their adult life as well. Management strategies for these conditions need to encompass broad multidisciplinary approaches, with an emphasis upon optimal care in the short-term and enhanced outcomes in the long-term. Whilst providing consistent high-quality, child and family-focused care is important, the introduction of new treatments and the promise of future cure provide ongoing challenges. The inflammatory bowel diseases are examples of such conditions.

The inflammatory bowel diseases comprise Crohn disease (CD) and ulcerative colitis (UC), with some individuals initially categorized as IBD unclassified (1). Increasing rates of IBD have been seen in many areas of the world. Australasian data show greater than 10-fold increases in rates of CD and UC in recent years (2, 3). High rates have been seen in individuals migrating from the Indian subcontinent to industrialized countries, such as Canada (4). More recent data also show increased rates of IBD in locations where this condition was previously thought to be rare (5).

These changes in the patterns of IBD mean that many more children are being diagnosed and consequently require management. At present these conditions remain incurable. Optimization of managements, with focused multi-disciplinary care within IBD teams is essential to ensure enhanced outcomes. Given the multiple overlapping aspects of IBD in children it is not conceivable that a single practitioner could provide all the elements required for optimal care. Along with pediatric gastroenterologists, dietitians, IBD nurses, psychologists, and surgeons are all key members of an IBD team.

Interventions are used in pediatric IBD to induce and then maintain remission. The enhancement or refinement of older, established therapies is important to maximize the efficacies of these approaches. This includes defining the best ways to use particular drugs such as aminosalicylates and thiopurines. It also includes increasing recognition of the important role that exclusive enteral nutrition plays in the management of active CD (6). However, the development and assessment of new therapies also remains critical. The therapeutic challenges include considering the best treatment at the right time, and developing individualized approaches for particular patients.

In recent years there has been a dramatic expansion in IBD-related knowledge. The development of new technologies has lead to huge advances in our understanding of the role of genetic mutations in the pathogenesis of IBD (7). Furthermore, new molecular techniques, including high throughput sequencing, has permitted a new wave of studies focusing upon the gastrointestinal microflora, helping us to better understand the role of bacterial agents in the onset of gut inflammation (8). However, there remain many gaps in our knowledge and understanding, requiring ongoing focused research endeavors.

Many of the recent advances have reflected the combined efforts of many individuals and many groups working together. Collaborative endeavors will be absolutely critical in the coming years, to ensure ongoing acquisition of new knowledge and understanding. Similar collaborative measures will also be essential to ensure that any new developments translate to improved care and enhanced outcomes.

## Evolving conditions

A number of conditions commonly cared for by pediatric gastroenterologists have evolved and changed in recent years: physicians must be able to adapt, learn and change with the new developments. Eosinophilic esophagitis and coeliac disease are just two examples.

Over the last couple of decades there have been huge changes in our approach to oesophageal eosinophilia, with the rise and rise of eosinophilic oesphagitis so that this is now an increasingly common entity. A landmark report was published late last century reporting a group of 10 young children presenting with upper gut symptoms associated with marked oesophageal eosinophilia that was unresponsive to acid suppression (9). The introduction of hypoallergenic formulae led to prompt improvement in symptoms and/or resolution of the mucosal eosinophilia. Subsequently, many centers have reported large increases in the prevalence of this condition. Management approaches have evolved, with the guidance of key consensus statements (10). However, there remains much to learn about this condition, especially with regards the long-term outcomes and optimal management approaches.

In recent years there have been a number of changes relating to coeliac disease: these will continue to challenge us in the coming years. The presentation patterns of this condition have changed. More and more children are now diagnosed with atypical symptoms or maybe no symptoms at all, with fewer children diagnosed with classical features of malabsorption (11). This in part relates to the advent of screening practices in settings of increased risk, such as Down syndrome and type 1 diabetes mellitus (12).

Increasing rates of coeliac disease have been noted in several areas of the world. Data from Finland suggest that the incidence of coeliac disease in that country is now almost 2% (13), whilst annual rates of diagnosis in children in one pediatric center in New Zealand have increased more than 10-fold in the last 15 years (Day et al., unpublished observations).

In addition, our understanding of the pathogenesis of coeliac disease has expanded greatly. New diagnostic tools have been developed and assessed. Very recently, a new approach to the diagnosis of coeliac disease has emerged (14). These protocols developed in Europe encompass clinical and biochemical assessments to reach a diagnosis, with exclusion of a requirement for endoscopic biopsies in many children. However, the application of these approaches may not be suitable in other areas of the world, such as Australasia and North America. Further focused studies are required to ensure that such protocols are universally appropriate. Clearly achieving a robust and clear diagnosis is important: the use of non-invasive tests to establish this has many potential advantages, including cost, resource implications, and parental distress. None-the-less, the development of improved and enhanced diagnostic approaches should lead to earlier and more effective diagnosis of this condition in children.

Furthermore, as we understand more about the pathogenesis of coeliac disease, it has become clear that there are a number of potential strategies by which these pathways could be interrupted (15). One very promising example is the development of vaccine-based treatment currently undergoing clinical trials in several countries (www.immunsant.com). This novel immune-based strategy promises to lead to a new approach to the treatment of coeliac disease. Namely, the introduction of a vaccine will mean that individuals diagnosed with coeliac disease who are then treated with the vaccine, will subsequently be able to tolerate a gluten-containing diet, without need for gluten-free diet. This promise of a cure for coeliac disease could transform the limitations of coeliac disease, and make huge differences in the lives of many children and families. However, this approach is still the “ambulance at the bottom of the cliff.” The development of effective and safe preventative strategies may be even more important and have greater impact. One example of such an approach is the timing of introduction of gluten-containing foods in infancy (16). Introduction of these cereals during breast-feeding in mid-infancy appears to the best approach, but further studies are focusing upon this.

Although new diagnostic protocols may facilitate quicker diagnosis of coeliac disease in children, a number of aspects of this condition remain unclear and require further study. It is likely that coeliac disease will continue to provide challenges for pediatric gastroenterologists in the future.

## Societal changes leading to new challenges

Several current social changes also impact greatly and provide further important challenges to pediatric gastroenterologists. Increasing rates of childhood obesity have been seen in many parts of the world (17). Although initially thought of as a consequence of the developed world, increasing rates are also now seen in developing countries.

Higher rates of childhood obesity have led to increased rates of non-alcoholic fatty liver disease (18). These changes have significant implications during childhood and also for future adult years. This condition has become an increasingly common indication for liver transplant, which consequently leads to a further set of long-term health issues.

## Workforce challenges

The changing patterns and increasing patient load consequent to many of the developments mentioned above have potential huge implications for resources in many countries around the world. Several workforce assessments have been conducted in North America: these estimate a workforce of around nine pediatric gastroenterologists per million children at that time (19, 20). Variations were seen between states in the USA and provinces in Canada. The current workforce in NZ is half of that seen in North America (4.5 pediatric gastroenterologists/million children), with consequent workload implications. However, the numbers of physicians need to be balanced also with other key staff providing care for these children—including dietitians and psychologists.

In some parts of the world, pediatric gastroenterology services have been covered by adult gastroenterology services or by general pediatricians. Dedicated and well-trained personnel ready to face the challenges faced by pediatric gastroenterologists, and able to provide high-quality child and family-friendly services are essential.

Endoscopy services are a particular illustration of the importance of this important concept. Pediatric endoscopy services should be provided by endoscopists with specific training in pediatric conditions and approaches (21). Children are not just little adults and reflect different conditions and different manifestations. Child friendly facilities are a further important aspect—not just for pre-schoolers with play-areas, but also for adolescents. The benefits of these considerations have not been illustrated in an evidence based fashion, but do appear implicit and self-evident.

## Conclusions

Many issues face pediatric gastroenterology now and many more will face the discipline in the coming years. Pediatric gastroenterology services include conditions affecting the length and breadth of the gut, along with impact upon many other systems. The spectrum of conditions managed and the incidence of many key conditions are both changing rapidly and dramatically: many of these changes are expected to continue apace. Pediatric gastroenterologists must be equipped with the skills and opportunities to be able to face and overcome these present challenges as well as the new challenges around the corner.

Numerous clinical, outcomes and laboratory-based research endeavors are now required to answer the many outstanding questions and to provide opportunities to overcome these challenges. Collaborative and multi-center population-based initiatives will be required to advance understanding of the epidemiology and natural history of many pediatric gastroenterology conditions. The development and assessment of new diagnostic tools and markers would ensure optimization of initial assessment and on-going management. Furthermore, other endeavors should focus on advancing our understanding of the pathogenesis of specific conditions, providing impetus to finding cures. In conclusion, together, we must take up the call to answer these key questions so that we can overcome the challenges facing us.
